# Key Principles for the Intergovernmental Science–Policy Panel on Chemicals and Waste

**DOI:** 10.1021/acs.est.2c08283

**Published:** 2023-01-30

**Authors:** Marlene Ågerstrand, Kenneth Arinaitwe, Thomas Backhaus, Ricardo O. Barra, Miriam L. Diamond, Joan O. Grimalt, Ksenia Groh, Faith Kandie, Perihan Binnur Kurt-Karakus, Robert J. Letcher, Rainer Lohmann, Rodrigo O. Meire, Temilola Oluseyi, Andreas Schäffer, Mochamad Septiono, Gabriel Sigmund, Anna Soehl, Temitope O. Sogbanmu, Noriyuki Suzuki, Marta Venier, Penny Vlahos, Martin Scheringer

**Affiliations:** † Department of Environmental Science, Stockholm University, 10691 Stockholm, Sweden; ‡ GEOMAR Helmholtz Centre for Ocean Research Kiel, 24148 Kiel, Germany; § Department of Biological and Environmental Sciences, University of Gothenburg, 40530 Gothenburg, Sweden; ∥ Faculty of Environmental Sciences and EULA Chile Centre, University of Concepcion, Concepción 4070386, Chile; ⊥ University of Toronto, Toronto, ON M5S 1A1, Canada; # Department of Environmental Chemistry, IDAEA-CSIC, 08034 Barcelona, Catalonia, Spain; @ Eawag, Swiss Federal Institute of Aquatic Science and Technology, 8600 Dübendorf, Switzerland; ∇ Department of Biological Sciences, Moi University, 30100 Eldoret, Kenya; ● Department of Environmental Engineering, Faculty of Engineering and Natural Sciences, Bursa Technical University, 16310 Yıldırım/Bursa, Turkey; ○ Departments of Chemistry and Biology, Environment and Climate Change Canada, National Wildlife Research Centre, Carleton University, Ottawa, ON KIS 5B6, Canada; ■ Graduate School of Oceanography, University of Rhode Island, Narragansett, Rhode Island 02881, United States; □ Universidade Federal do Rio de Janeiro, Campus Duque de Caxias, Santa Cruz da Serra, Rio de Janeiro 25240-005, Brazil; ▲ Department of Chemistry, University of Lagos, Akoka, Lagos 101017, Nigeria; △ Institute for Environmental Research, RWTH Aachen University, 52074 Aachen, Germany; ▼ Nexus3 Foundation, Denpasar 80223, Bali, Indonesia; ▽ Department of Environmental Geosciences, Centre for Microbiology and Environmental Systems Science, University of Vienna, 1090 Vienna, Austria; ⬢ International Panel on Chemical Pollution, 8044 Zürich, Switzerland; ⬡ Ecotoxicology and Conservation Unit, Department of Zoology, Faculty of Science, University of Lagos, Akoka, Lagos 101017, Nigeria; ◆ National Institute for Environmental Studies, 16-2 Onogawa, Tsukuba 305-8506, Ibaraki, Japan; ◇ O’Neill School of Public and Environmental Affairs, Indiana University, Bloomington, Indiana 47405, United States; ⬟ Marine Sciences, University of Connecticut, Groton, Connecticut 06340, United States; ⬠ Institute of Biogeochemistry and Pollutant Dynamics, ETH Zürich, 8092 Zürich, Switzerland; ☆ RECETOX, Masaryk University, 625 00 Brno, Czech Republic

**Keywords:** chemical pollution, human health, environmental health

## Introduction

In 2021, the United Nations Environment Programme (UNEP) recognized chemical pollution as a planetary crisis tantamount to climate change and biodiversity decline.^1^ In an important next step, the international community agreed in March 2022 on establishing an independent, intergovernmental science–policy panel on chemicals, waste, and pollution prevention (hereafter termed “the Panel”).^2^ This Panel will take its place among two other intergovernmental bodies, the Intergovernmental Panel on Climate Change (IPCC)^3^ and the Intergovernmental Science-Policy Platform on Biodiversity and Ecosystem Services (IPBES).^4^ Now is a crucial time for establishing the Panel, following a process facilitated by UNEP to negotiate the Panel’s scope, functions, and institutional design, with the ambition to formally establish the Panel in 2024.

As a group of international scientists working on chemical pollution, we applaud this milestone of progress to initiate the establishment of a panel for chemicals, waste, and pollution prevention. At the beginning of the negotiating process, we would like to highlight the following 10 critical aspects for consideration in determining the settings of the Panel.

### 1 Why a New Panel?

The new Panel is needed to fill a critical gap pertaining to the mounting and accelerating impacts on human and environmental health caused by chemical pollution and waste globally. The highly multifaceted and heterogeneous impacts of chemical pollution encompass a wide array of issues that often show dynamic development and require international action.^5^ Therefore, there is an urgent need for a big-picture perspective resulting from a comprehensive and ongoing horizon scanning, monitoring, interpretation of data, and synthesis of individual findings. This goes beyond the remit of existing bodies at the national, regional, and international levels because their scopes and mandates are limited to certain chemicals, geographical areas, or jurisdictions (examples here include the Basel, Stockholm, Rotterdam, and Minamata conventions). For the development of effective action at the global level, comprehensive and authoritative scientific assessments are crucial.

### 2 Avoid Paralysis by Analysis

Current knowledge is sufficient for several chemicals or groups of chemicals with hazardous properties (for example, lead, mercury, asbestos, and several pesticides) to enable implementing evidence-based solutions. As an integrator of scientific information, the Panel must avoid “paralysis by analysis” by repeatedly re-assessing the same topics and substances. The reports “Late lessons from early warnings I & II”^6,7^ provide a wide range of examples (e.g., benzene, DDT, mercury, lead, asbestos, and PCBs) where continued research expanded and deepened the understanding of the issue but largely confirmed earlier insights and where, accordingly, action could and should have been taken earlier. Also, knowledge of the proper treatment of many types of waste is available, which helps to reduce and ideally prevent the spread of many pollutants into the environment. The Panel should therefore provide a comprehensive bigger picture and forward-thinking reviews and assessments to enable horizon scanning in order to identify new research gaps and needs, also for more recently identified “emerging” issues.

### 3 Scope

The Panel’s work needs to be broad and inclusive to properly respond to the breadth and complexity of global chemical production, use, releases, and disposal, involving up to several hundred thousand chemicals, of which a substantial fraction is hazardous to humans and/or ecosystem health. This includes well-characterized “legacy” chemicals, but also a much larger array of “chemicals of emerging concern” and novel waste streams such as non-agricultural biocides, per- and polyfluoroalkyl substances (PFAS), pharmaceuticals; personal care products, toxic metal(loid)s, rare-earth elements, and other chemicals being used in growing renewable energy and digital technology industries.^8^ In addition to understanding the impact of the use of single substances, effects from mixtures also need to be considered.^9^ Also to be addressed are the numerous (and often insufficiently known) impurities in the produced chemicals and the even larger array of (potential) chemical contaminants resulting from abiotic and biotic environmental transformation processes.^10^

### 4 Tasks and Outputs

As with other science–policy bodies, the Panel should be policy-relevant but not policy-prescriptive in providing integration and analyses that support sound, evidence-based policy development. Early warning of new and emerging chemical contamination issues, including the analysis of options for policy actions, should be among the regular tasks for the Panel. The Panel should inform, and provide feedback to, policymakers, especially in national and multinational jurisdictions and regulatory bodies, about important scientific gaps and findings and also inform the scientific community about policy-relevant scientific questions. Chemical hazard and risk assessments are performed by existing bodies, and while these processes could be improved, assessments of individual chemicals should not be the primary focus of the Panel. Rather, the Panel should address strategically important and broader issues. These issues may include the identification and assessment of chemical groups of concern (see above), effective strategies for avoiding regrettable substitution, etc.

### 5 Roles of Different Actors

The models of IPCC and IPBES should inspire and inform the Panel in terms of the roles and responsibilities of various societal actors and groups contributing to its work. Governments should nominate independent experts as the main “workforce” of the Panel. Independent scientists will provide primary scientific results; independent scientists can also facilitate the appropriate use of the assessments of the Panel. Also, national and international government scientists should be included as independent experts. However, while government scientists are free to publish scientific findings in the peer-reviewed literature, they may not be permitted to comment on or contradict existing government policies on chemicals, specifically if there is divergence or conflict of opinion. Other actors such as intergovernmental organizations, environmental nongovernmental organizations, civil society groups, community-based organizations, and the private sector should act as observers and be invited to provide input.

### 6 Conflicts of Interest

The Panel needs to establish and enforce a strict conflict-of-interest policy. In particular, it must be recognized that, while the private sector might be privy to important information, its representatives may also have inherent conflicts of interest. Research shows that systematic strategies for influencing, and in some cases discrediting, both the science and the subsequent policy development have been used by industries manufacturing tobacco, coal, sugar, oil, and chemicals.^11^ This need for a clear conflict-of-interest statement also applies to those who act through nongovernmental bodies such as multisector associations. All experts contributing to the work of the Panel must declare their conflicts of interest, financial and otherwise.

### 7 Data

Following IPCC and IPBES, a range of data from the peer-reviewed scientific literature, gray literature, and existing biological and chemical monitoring programs will be foundational for the outcomes of the Panel. This highlights the importance for the Panel to make use of all available reliable and relevant data and not to limit itself to prescribed data formats such as those defined by specific test guidelines or good laboratory practice (GLP), as such a limitation may exclude studies of significance, especially from all geographic areas. Studies from the scientific literature have proven to be valuable in the restriction of chemicals under the EU chemicals regulation, REACH, meaning that these studies were considered of sufficient reliability and relevance.^12^ As a model, the new Transparency Regulation in the European Union’s food laws is also expected to open up previously unavailable data for use in other domains of chemical oversight in the EU and beyond. Transparency of data and the Panel’s workflow is crucial. Furthermore, the inclusion of under-resourced countries or regions in mobilizing data (digitized and undigitized) should be fostered.

### 8 Establish Inclusive Knowledge Exchange

The production, use, and disposal of chemicals are important parts of human activities across the globe. However, different countries and regions approach the management of hazardous chemicals and waste in different ways, driven by different government political climates, economic systems, and state capacity, as well as different geographical and ecological conditions. It will therefore be critical that the Panel apply a transdisciplinary approach to the integration of knowledge, across different temporal and spatial scales and different regions of the world. The ability of the Panel to provide useful options for improving the management of chemicals and waste will depend on an integration of natural and biomedical sciences [e.g., chemistry, exposure sciences, (eco)toxicology, ecology, epidemiology, and risk assessment], social sciences (e.g., economics, political sciences, communication sciences, and law), humanities, traditional and local indigenous knowledge, and other knowledge sources. The adequate involvement of experts in risk communication will be critical for the dissemination and contextualization of the Panel’s work.

### 9 Work Program and Uptake of Results

The Panel has the potential to contribute objective, authoritative, and current syntheses of information for countries to utilize as they develop national and international policies. The work of the Panel will provide a new and valuable resource from which information can be drawn and from which suggestions for future actions can be developed. Again, we emphasize that the outcomes of the Panel will be policy-relevant, but not policy-prescriptive, and the actual way forward will be decided by political processes at national, regional, and international levels that feed into the intergovernmental process.

### 10 Global Representation and Participation

The Panel needs to ensure that all countries are adequately represented in terms of experts and their backgrounds, data, and access to information. The Panel should operate on a global scale to identify and address critical needs in terms of both information and capacity. One key task of the Panel is to address the limitation that currently available data and expertise are centered on high-income countries, even though the majority of pollution-related impacts and deaths occur in low- and middle-income countries. The Panel should recognize that these countries, broadly defined as the “Global South”, have a critical but poorly recognized role in the production of chemicals and handling of chemical waste and carry a substantial part of the burden from chemical pollution, while at the same time being global biodiversity hotspots.

## Join the Process

Academic scientists around the world will play a crucial role in the work of the Panel. The time to prepare the ground for this important task is now, by providing input to the political process defining the setup of the Panel. We encourage scientists from all countries to join the discussion, indicate their interest in the Panel’s work, identify urgent chemical pollution and waste issues in their regions, and share their insights with fellow scientists as well as policymakers nationally and internationally.

An Open-Ended Working Group (OEWG) has been established under UNEP as the venue where the scope, function, and structure of the Panel are being discussed and, eventually, will be decided.^13^ Decisions will be made by governments; nongovernmental stakeholders can be accredited as observers to contribute to the discussion. Academic scientists can join this process through several avenues. One is by being nominated as a member of their government’s delegation. This will be an important role for scientists to play but will be limited to a relatively small number of scientists per country.

Another possibility is through accredited nongovernmental organizations (NGOs). However, many of these organizations may have specific mandates that do not necessarily reflect, or are even in contradiction with, the role of independent scientists. Therefore, there is a need for organizations that fulfill the requirements for accreditation (most importantly, nongovernmental, not-for-profit status, engagement in the field of environment, and international scope) and that can offer a platform for academic scientists to present their contributions to the discussion. This function may be fulfilled by scientific societies such as the Royal Society of Chemistry (RSC), the Society of Environmental Toxicology and Chemistry (SETAC), the Endocrine Society, and others. An example of an organization that specifically and exclusively represents academic scientists is the International Panel on Chemical Pollution (IPCP).

Finally, academic institutions may also be accredited with UNEP, which would facilitate participation of their faculty members. However, very few universities have so far applied for accreditation, and it is not yet fully clear under which circumstances universities fulfill the status of “nongovernmental”. According to UNEP, applications from academic institutions are welcome, also to further clarify the accreditation process for universities.^14,15^

The development of the Panel represents an important step forward. It recognizes the global nature and the complexity of chemicals and waste issues. The Panel will provide the much-needed forum for comprehensively addressing these challenges.

## References

[ref1] United Nations Environment Programme. Making Peace with Nature: A scientific blueprint to tackle the climate, biodiversity and pollution emergencies. Nairobi, 2021. https://www.unep.org/resources/making-peace-nature (accessed 2022-11-05).

[ref2] United Nations Environment Assembly. Resolution 5/8 adopted by the United Nations Environment Assembly on 2 March 2022: Science-policy panel to contribute further to the sound management of chemicals and waste and to prevent pollution. Nairobi, 2022. https://www.unep.org/resources/resolutions-treaties-and-decisions/UN-Environment-Assembly-5-2 (accessed 2022-11-05).

[ref3] Intergovernmental Panel on Climate Change. https://www.ipcc.ch/ (accessed 2022-11-05).

[ref4] Intergovernmental Science-Policy Platform on Biodiversity and Ecosystem Services. https://ipbes.net/ (accessed 2022-11-05). 10.1007/s10531-021-02261-0 PMC8313413 34334969

[ref5] United Nations Environment Programme. Global Chemicals Outlook II - From Legacies to Innovative Solutions: Implementing the 2030 Agenda for Sustainable Development. Nairobi, 2019. https://www.unenvironment.org/resources/report/global-chemicals-outlook-ii-legacies-innovative-solutions (accessed 2022-11-05).

[ref6] European Environment Agency. Late Lessons from Early Warnings: the Precautionary Principle 1896–2000; Environmental Issue Report 22. Copenhagen, 2001. https://www.eea.europa.eu/publications/environmental_issue_report_2001_22/at_download/file.

[ref7] European Environment Agency. Late Lessons from Early Warnings: Science, Precaution, Innovation; EEA Report 1/2013. Copenhagen, 2013. http://www.eea.europa.eu/publications/late-lessons-2.

[ref8] Müller L. K.; Ågerstrand M.; Backhaus T.; Diamond M. L.; Erdelen W. R.; Evers D.; et al. Policy options to account for multiple chemical pollutants threatening biodiversity. Environ. Sci. Adv. 2023, 10.1039/D2VA00257D.

[ref9] Kortenkamp A.; Backhaus T.; Faust M. State-of-the-Art Report on Mixture Toxicity. Brussels, 2009. https://ec.europa.eu/environment/chemicals/effects/pdf/report_mixture_toxicity.pdf.

[ref10] Escher B. I.; Fenner K. Recent Advances in Environmental Risk Assessment of Transformation Products. Environ. Sci. Technol. 2011, 45, 3835–3847. 10.1021/es1030799. 21473617

[ref11] Goldberg R. F.; Vandenberg L. N. Distract, delay, disrupt: examples of manufactured doubt from five industries. Rev. Environ. Health 2019, 34, 349–363. 10.1515/reveh-2019-0004. 31271562

[ref12] Borchert F.; Beronius A.; Ågerstrand M. Characterisation and analysis of key studies used to restrict substances under REACH. Environ. Sci. Europe 2022, 34, 83 10.1186/s12302-022-00662-8.

[ref13] UNEP. Open-Ended Working Group for the preparation of proposals for the science-policy panel on chemicals, waste and pollution. 2023. https://www.unep.org/events/conference/oewg1-science-policy-panel-contribute-further-sound-management-chemicals-and (accessed 2023-01-06).

[ref14] Personal communication by Alexander Juras, Chief, UNEP’s Civil Society Unit, January 8, 2023.

[ref15] United Nations Environment Programme. Accreditation Flyer in English. https://wedocs.unep.org/handle/20.500.11822/20737 (accessed 2023-01-06).

